# Evaluation of factors associated with the difficulty in finding receiving hospitals for traffic accident patients at the scene treated by emergency medical services: a population‐based study in Osaka City, Japan

**DOI:** 10.1002/ams2.291

**Published:** 2017-06-16

**Authors:** Yusuke Katayama, Tetsuhisa Kitamura, Kosuke Kiyohara, Taku Iwami, Takashi Kawamura, Sumito Hayashida, Hiroshi Ogura, Takeshi Shimazu

**Affiliations:** ^1^ Department of Traumatology and Acute Critical Medicine Osaka University Graduate School of Medicine Suita Japan; ^2^ Division of Environmental Medicine and Population Sciences Department of Social and Environmental Medicine Graduate School of Medicine Osaka University Suita Japan; ^3^ Department of Public Health Tokyo Women's Medical University Tokyo Japan; ^4^ Kyoto University Health Services Kyoto Japan; ^5^ Osaka Municipal Fire Department Osaka Japan

**Keywords:** Difficulty in hospital acceptance, emergency medical services, prehospital care, prolongation of transport time, traffic accident

## Abstract

**Aim:**

Although the prolongation of the time between injury and hospital arrival of traffic accident patients can influence their prognosis, factors associated with the difficulty in hospital acceptance of these patients have not been sufficiently evaluated in Japan.

**Methods:**

We retrospectively analyzed the population‐based ambulance records of all traffic accident patients for whom the Osaka Municipal Fire Department (Osaka City, Japan) dispatched an ambulance in 2013. We defined “cases with difficulty in hospital acceptance” as cases that required ≥4 calls by emergency medical service personnel at the scene before receiving hospital acceptance. We included patient characteristics (age, sex, coma status, and trauma severity judged by emergency medical service personnel), time factors (day/night or weekday/holiday and weekends), and accident location for multivariable logistic regression analysis to assess factors associated with the difficulty in hospital acceptance.

**Results:**

Among 13,427 traffic accident patients, 2,033 (15.1%) were cases with difficulty in hospital acceptance. Pediatric patients (adjusted odds ratio [OR], 1.265; 95% confidence interval [CI], 1.060–1.509), male sex (adjusted OR, 1.260; 95% CI, 1.135–1.398), moderate‐grade trauma (adjusted OR, 2.241; 95% CI, 1.972–2.547), severe‐grade trauma (adjusted OR, 2.057; 95% CI, 1.249–3.388), holidays and weekends (adjusted OR, 1.702; 95% CI, 1.539–1.882), and night‐time (adjusted OR, 2.720; 95% CI, 2.443–3.027) were positively associated with difficulty in hospital acceptance.

**Conclusions:**

Using population‐based ambulance records from a large urban community in Japan, we showed that the difficulty in hospital acceptance of patients at the scene of traffic accidents was positively associated with several prehospital factors.

## Introduction

Recently, the number of traffic accidents has been decreasing in Japan because of drivers’ observance of traffic laws, advances in automotive technology, and crackdowns for traffic violations by the police.^1^ In accordance with this decrease, the number of casualties from traffic accidents has been also decreasing,^1^ and it is smaller in Japan than in other countries.^2^ In comparison with statistics in many countries of Europe, Australia and North America, however, the proportion of accidents in Japan involving pedestrians, cyclists, and the elderly ≥65 years old is larger.^3^

In Japan, when an emergency medical service (EMS) is activated by emergency telephone call, the EMS personnel dispatched at the scene select the appropriate hospital after assessing the patient condition. Then, after obtaining the permission from the selected hospital to receive the patient through a telephone call (this permission was defined as hospital acceptance in this study), the ambulance transports the patient to the hospital.^4^, ^5^ Although prolongation of the time between injury and hospital arrival can worsen the prognosis of traffic accident patients,^6^, ^7^ it is unclear what types of prehospital factors influence the hospital selection of EMS personnel and the hospital's acceptance of patients at the scene of a traffic accident.

The Osaka Municipal Fire Department has been collecting records of ambulance dispatch in Osaka City, Japan, a metropolitan community with approximately 2.6 million residents and over 200,000 emergency dispatches every year. In this study, we hypothesized that patient characteristics such as age, sex, disturbance of consciousness, the severity of injury, accident location, time of day, and day of the week would influence hospital acceptance of traffic accident patients. By reviewing a large number of ambulance records in Osaka City, we assessed the relationship of these prehospital factors with hospital acceptance of traffic accident patients.

## Methods

### Study design, population, and setting

This study was a retrospective observational study carried out from 1 January, 2013 through to 31 December, 2013 using ambulance records from Osaka City. We registered all traffic accident patients transported by EMS personnel from the scene to hospital in this study. We excluded emergency patients who were either not transported or were transported to hospitals requested by the patients and/or their family and those undergoing interhospital transport. Generally, ambulance records are considered as governmental documents, and the acquisition of patients’ informed consent was waived because researchers dealt only with anonymous data not linkable to the patients. No personal data was accessed. This study was approved by the Ethics Committees of Osaka University Graduate School of Medicine (Suita, Japan) and Kyoto University Graduate School of Medicine (Kyoto, Japan).

### Emergency medical service system and hospitals in Osaka City

The EMS in Japan is a public service, and patients are transported to hospital by the public EMS system. Osaka City, the largest metropolitan community in western Japan, had a population of about 2.6 million in 2013. The EMS system is operated by the Osaka Municipal Fire Department and is activated by phoning 119. In 2013, there were 25 fire stations with 60 ambulances and one dispatch center in Osaka City. Emergency medical service life support is provided 24 h a day, 7 days a week. Usually, each ambulance has a crew of three EMS personnel including at least one paramedic, and the annual number of patients transported to hospitals by EMS in this area is approximately 200,000. Osaka City had a total of 184 hospitals with 32,662 beds in 2014,^8^ and 94 of them, including six critical care centers, are to accept emergency patients from ambulances. In Osaka City, emergency dispatchers do not make phone calls to hospitals to determine patient acceptance. Rather, using the protocol established by the Osaka Municipal Fire Department, EMS personnel at the scene select an appropriate hospital near the scene that is best able to treat the emergency patient according to medical urgency or the patient's condition.

### Data collection and quality control

Data were uniformly collected using specific data collection forms and included age, sex, Glasgow Coma Scale score, location of call, the severity of injury, chronological factors such as time of the day and day of the week, time‐course of transport, such as time of the call, time of contact with the patient, and time of hospital arrival, and the number of phone calls made to hospitals by EMS personnel.

Emergency medical service personnel recorded these data in cooperation with the physicians caring for the patients and then transferred the data to the information center in the Osaka Municipal Fire Department. If the data sheet was incomplete, information center personnel returned it to the responsible EMS personnel so they could complete it.

### End‐point

The main study end‐point was the determination of factors associated with the difficulty in hospital acceptance at the scene. Based on the definition of the Fire and Disaster Management Agency of Japan,^9^ difficulty in hospital acceptance at the scene is defined when EMS personnel need to make ≥4 telephone calls to obtain permission for patient reception.

### Statistical analysis

Patient and EMS characteristics between the two groups (<4 and ≥4 phone calls) were evaluated by χ^2^‐test for categorical variables and Wilcoxon tests for continuous variables. In addition, we assessed the correlation between the time interval from call to contact with a patient and the number of phone calls. We calculated the odds ratio (OR) and 95% confidence interval (CI) with use of a logistic regression model to evaluate the factors associated with the difficulty in hospital acceptance at the scene for traffic accident patients, and we considered potential factors that existed before the EMS personnel made contact with the emergency patient. These factors included age group (children aged <15 years old, adults aged 15–64 years old, and elderly aged ≥65 years old), sex, disturbance of consciousness (defined as Glasgow Coma Scale ≤8), the severity of injury according to the definition of the Ministry of Health, Labor and Welfare of Japan for paramedics (mild, moderate, severe, or cardiopulmonary arrest [CPA] at the scene),^9^ accident location (road, highway, train, train station or railroad, and others such as private road and parking lot), time of day (daytime or night‐time), and day of the week (weekday or holidays and weekends).

All tests were two‐tailed, and *P*‐values <0.05 were considered statistically significant. Statistical analyses were carried out using spss statistical package version 22.0J (IBM, Armonk, NY, USA).

## Results

**Figure 1:**
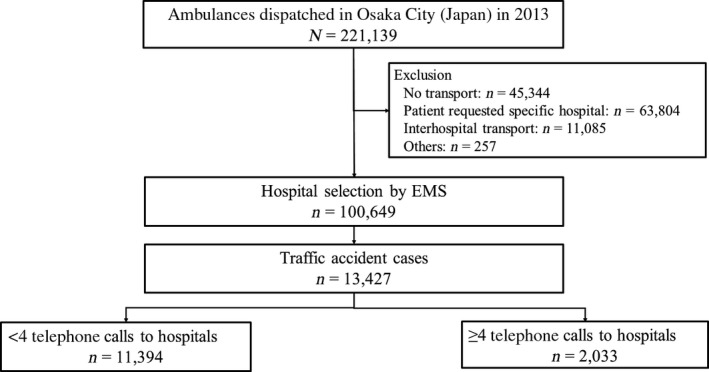
Patient flow in this study of traffic accident patients for whom the Osaka Municipal Fire Department (Osaka City, Japan) dispatched an ambulance in 2013.

**Figure 2:**
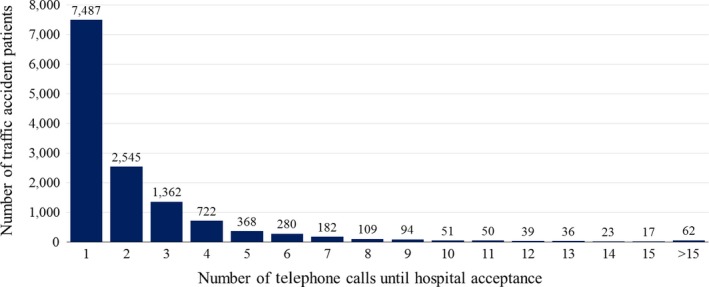
Histogram of the number of telephone calls made to hospitals by emergency medical service personnel at the scene of traffic accidents to request patient acceptance (Osaka City, Japan, 2013).

During the study period, 221,139 EMS dispatches were made in Osaka City. Of them, 100,649 patients were enrolled in this study. The following cases were excluded: 45,344 patients were not transported by ambulances; 63,804 patients were transferred to hospitals according to the request of the patient or their family; 11,085 patients were transported between hospitals; and 257 patients had other reasons. The number of emergency patients injured by traffic accidents was 13,427, and these were the subjects of this study (Fig. 1). Among these patients, ≥4 calls were made to hospitals by EMS personnel at the scene before receiving hospital acceptance for 2,033 traffic accident patients. The median number of calls was 1 (interquartile range, 1–3), and ≥10 phone calls to hospitals were made by EMS personnel for 278 patients (2.1%) (Fig. 2).

**Figure 3:**
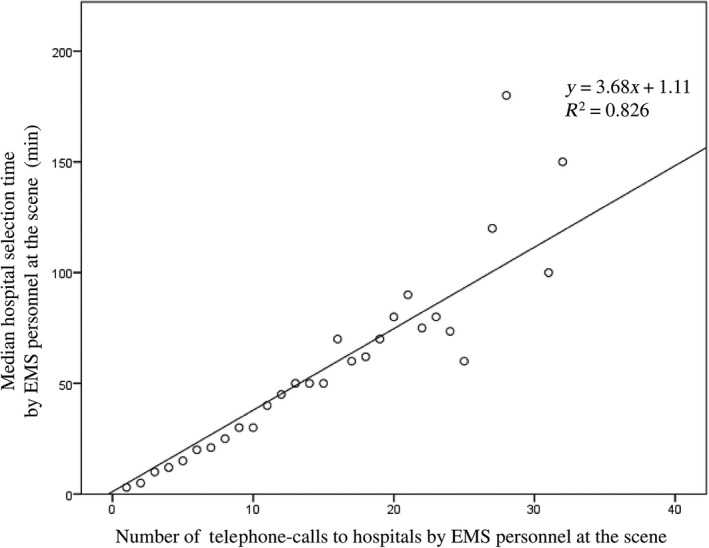
Correlation of the interval from ambulance dispatch to hospital arrival and the number of telephone calls to hospitals by emergency medical service (EMS) personnel to request patient acceptance following traffic accidents (Osaka City, Japan, 2013).

Characteristics of the traffic accident patients with <4 or ≥4 phone calls to hospitals by EMS personnel until hospital acceptance was made are shown in Table 1. Compared with patients requiring <4 phone calls, of those requiring ≥4 phone calls, there were more male patients, more with moderately severe injury, and more associated with accidents that occurred on holidays and weekends and during night‐time; these differences are statistically significant. Although the time interval from EMS call to contact with a patient was similar between the two groups, the time interval from EMS call to hospital arrival for patients requiring ≥4 phone calls was significantly longer than that for those requiring <4 phone calls (median, 27 versus 49; *P* < 0.001). The time interval from call to hospital acceptance correlated well with the number of phone calls made by EMS at the scene (y = 3.68x+1.11, R^2^ = 0.826) (Fig. 3).

**Table 1:** Patients and emergency medical service (EMS) characteristics by number of telephone calls to hospitals by EMS to request patient acceptance following traffic accidents (Osaka City, Japan, 2013)

	Number of calls to hospitals by EMS		*P*‐value
	<4	≥4	
	(*n* = 11,394)	(*n* = 2,033)	
Age, years, median (IQR)	40 (24–58)	40 (25–56)	0.349
Age group, *n* (%)			
Children aged <15 years	971 (8.5)	176 (8.7)	0.005*
Adults aged 15–64 years	8,344 (73.2)	1,556 (76.5)	
Elderly aged ≥65 years	2,079 (18.2)	312 (15.3)	
Male, *n* (%)	6,906 (60.6)	1,392 (68.5)	<0.001*
Disturbance of consciousness (GCS ≤8), *n* (%)	122 (1.1)	30 (1.5)	0.119
Severity of injury			
Mild	9,937 (87.2)	1,567 (77.1)	<0.001*
Moderate	1,338 (11.7)	435 (21.4)	
Severe	83 (0.7)	30 (1.5)	
CPA at the scene	36 (0.3)	1 (0.0)	
Location of occurrence, *n* (%)			
Road	10,691 (93.8)	1,862 (91.6)	0.002*
Highway	268 (2.4)	58 (2.9)	
Train	67 (0.6)	14 (0.7)	
Train station or railroad	38 (0.3)	13 (0.6)	
Other	330 (2.9)	86 (4.2)	
Time of day, *n* (%)			
Day (9.00 am–5.00 pm)	5,898 (51.8)	589 (29.0)	<0.001*
Night (5.00 pm–9.00 am)	5,504 (48.3)	1,448 (71.2)	
Day of the week, *n* (%)			
Weekday	8,095 (71)	1,233 (61)	<0.001*
Holidays and weekends	3,299 (29)	800 (39)	
Time from patient's call to contact by EMS, min, median (IQR)	5 (4–7)	5 (4–7)	0.052
Time from patient's call to hospital arrival, min, median (IQR)	27 (22–33)	49 (39–62)	<0.001*

CPA, cardiopulmonary arrest; GCS, Glasgow Coma Scale; IQR, interquartile range. *P <0.05

Factors associated with ≥4 phone calls to hospitals by EMS personnel at the scene before EMS personnel obtained a permission to transport the patient to the receiving hospital are shown in Table 2. Pediatric patients (adjusted OR, 1.265; 95% CI, 1.060–1.509), male sex (adjusted OR, 1.260; 95% CI, 1.135–1.398), moderate‐grade trauma (adjusted OR, 2.241; 95% CI, 1.972–2.547), severe‐grade trauma (adjusted OR, 2.057; 95% CI, 1.249–3.388), train station or railroad accident (adjusted OR, 2.103; 95% CI, 1.068–4.141), accidents occurring on holidays and weekends (adjusted OR, 1.702; 95% CI, 1.539–1.882), and accidents occurring at night (adjusted OR, 2.720; 95% CI, 2.443–3.027) were significantly associated with ≥4 phone calls to hospitals by EMS personnel. Conversely, CPA at the scene (adjusted OR, 0.138; 95% CI, 0.018–1.083) was unlikely to be associated with ≥4 phone calls to hospitals by EMS personnel.

**Table 2:** Factors associated with ≥4 telephone calls to hospitals by emergency medical service personnel to request patient acceptance following traffic accidents (Osaka City, Japan, 2013)

	Adjusted OR	95% CI		*P*‐values
Age group (versus adults aged 15–64 years)				
Children aged <15 years	1.265	1.060	1.509	0.009*
Elderly aged ≥65 years	0.875	0.760	1.006	0.061
Male sex (versus female)	1.260	1.135	1.398	<0.001*
Disturbance of consciousness (GCS ≤8)	0.910	0.551	1.503	0.712
Injury severity (compared with mild grade)				
Moderate	2.241	1.972	2.547	<0.001*
Severe	2.057	1.249	3.388	0.005*
CPA at the scene	0.138	0.018	1.083	0.060
Location of occurrence (comparison with road)				
Highway	1.183	0.879	1.591	0.267
Train	1.433	0.788	2.604	0.238
Station and railroad	2.103	1.068	4.141	0.031*
Other	1.461	1.137	1.877	0.003*
Night‐time (compared with daytime)	2.720	2.443	3.027	<0.001*
Holidays and weekends (compared with weekday)	1.702	1.539	1.882	<0.001*

CI, confidence interval; CPA, cardiopulmonary arrest; GCS, Glasgow Coma Scale; OR, odds ratio. *P <0.05

## Discussion

From the ambulance records of emergency patients in a large metropolitan city in Japan, we showed that prehospital factors such as children, male sex, moderate and severe grades of injury, train station and railroad as the location of occurrence, night‐time, and holidays and weekends were associated with difficulty in hospital acceptance of traffic accident cases. A detailed population‐based ambulance record enabled us to discover factors associated with difficulty in hospital acceptance of emergency patients in traffic accidents, and our findings will help to improve emergency medical responses for traffic accidents in Japan.

This study underscored that the factor of moderate or severe grade of injury was associated with difficulty in hospital acceptance, which suggests that a number of hospitals could not accept emergency patients with moderate and severe grades of injury because there are few hospitals that could treat such patients with severe injuries. Therefore, we need to change the system so that severely injured traffic accident patients are transported to hospitals smoothly and appropriately. However, CPA at the scene of the traffic accident was not associated with difficulty in hospital acceptance, which suggests that the protocol for transporting and receiving CPA trauma patients is separated from that for non‐CPA trauma patients in Osaka and the protocol requiring emergency medical centers to accept traffic accident patients with CPA at the scene was properly followed.

This study also showed that male sex was associated with difficulty in hospital acceptance. A previous study noted that male patients tended to be more severely injured than female patients in traffic accidents.^10^ Therefore, because male patients were expected to have more severe injuries from traffic accidents, hospitals might hesitate to accept them. Further studies would be needed to assess the gender differences not only in traffic accidents but also in other diseases requiring emergency treatment.

Chronological factors such as occurrence of traffic accidents at night and on holidays and weekends were also associated with difficulty in hospital acceptance. Although the number of automobiles moving during the night and on holidays and weekends is much the same as that at on weekdays,^11^ the number of hospital personnel is reduced during night‐time and on holidays and weekends in Japan. Indeed, survival from out‐of‐hospital cardiac arrests was reported to be poor during the night‐time and on holidays in Japan.^12^ In addition, traffic accidents occurring during holidays and weekends and at night are frequently associated with alcohol, drugs, and drowsy driving, and tend to involve severely injured victims, which may be related to the difficulty in finding hospitals to receive patients.^13^, ^14^, ^15^ Medical resource allocation is one of the important issues in an emergency medical system, and both medical institutions and local governments must arrange a system such that emergency patients can be transported and accepted without delay 24 h a day, 7 days a week.

This study also revealed that pediatric traffic accidents were associated with the difficulty in hospital acceptance. A previous study showed that pediatric pedestrians tended to be involved in traffic accidents more often than adult pedestrians.^10^ In Japan, usual patient care other than trauma is mainly carried out by an independent specialist treating a particular disease, even in the general hospitals, so there are few medical institutions that may be able to treat multiple trauma patients, especially children, who may require intensive assessment and care from a multidisciplinary approach. In addition, children often cannot appropriately express their symptoms and signs to EMS personnel or parents, which might be one of the factors associated with the difficulty in hospital acceptance. It is notable that dedicated pediatric acute care centers have not been adequately established in Japan compared with other developed countries. Therefore, it is necessary that sufficiently dedicated pediatric services are provided to resolve the problem shown in this study.

### Limitations

This study has some limitations. An important limitation of this study is that we did not obtain in‐hospital information about detailed diagnosis, medical treatment, and the patients’ prognosis after hospital arrival. Therefore, we could not assess whether the difficulty in hospital acceptance affected patient outcome after hospital arrival. After October 2014, we began to collect data including emergency patients’ information such as in‐hospital outcomes and treatments and will address this issue in the near future.^16^ Additionally, because this was an observational study, there might be unknown confounding factors that influenced our results.

## Conclusion

From the population‐based ambulance records of a large urban community, we showed that several prehospital factors were positively associated with the difficulty of hospital acceptance of traffic accident patients at the scene. On the basis of this study, we believe that the development of emergency medical systems that ensure the establishment of emergency medical centers with dedicated pediatric services and a rotation system for hospitals to accept patients at night will lead to improved outcomes among traffic accident patients in Japan.

## Disclosure

Conflict of Interest: None declared.
